# Cancer Cell Dormancy in the Bone Microenvironment

**DOI:** 10.1007/s11914-025-00934-1

**Published:** 2025-10-15

**Authors:** Chloe J. Harris, Georgia R. Stewart, Abigail Foston, Alanna C. Green

**Affiliations:** Mellanby Centre for Musculoskeletal Research and Healthy Lifespan Institute, School of Medicine and Population Health, Faculty of Health, University of Sheffield, Sheffield, S10 2RX UK

**Keywords:** Bone metastasis, Cancer cell dormancy, Senescence, Myeloma, Breast cancer, Prostate cancer

## Abstract

**Purpose of the Review:**

Cancer cell dormancy in the bone microenvironment presents a major obstacle to curative therapy across multiple cancer types. The bone harbours specialised pro-dormancy niches that promote the induction and long-term maintenance of dormant cancer cells. Many cancers originate in or metastasise to bone, but share the phenomenon of dormancy, which enables therapy evasion and later reactivation to cause disease relapse. This review provides recent updates in preclinical and clinical findings regarding dormancy in bone.

**Recent Findings:**

Studies have identified specific cell types including bone lining cells and Nestin + NG2 + MSCs as pro-dormancy niche cells. Newly identified signalling pathways, such as autophagy, have been found to support dormancy, with degrees of built-in redundancy. These advances have led to ongoing clinical trials in this space that mean new dormancy-targeting therapies, such as the autophagy inhibitor hydroxychloroquine, are on the horizon.

**Summary:**

This review explores extrinsic and intrinsic regulators of cancer cell dormancy in the bone microenvironment and highlights recent advances in development of therapies that can target cancer cell dormancy.

## Introduction

Cancer cell dormancy presents a major barrier to curative treatment of cancers that grow in the bone marrow microenvironment. This includes primary cancers like multiple myeloma, and also cancers that metastasise to bone, including breast and prostate cancer. Non-proliferative cells, whether dormant, senescent or quiescent, are largely resistant to therapies that target cycling cells. Dormant cancer cells are also relatively rare, making them challenging to target. Here we will discuss cancer cell dormancy, how it is regulated by the bone microenvironment and how recent progress in this area means that new treatments are on the horizon.

## Defining Dormancy, Senescence and Quiescence

In cancer, dormancy can refer to two separate phenomena, tumour mass dormancy or cellular dormancy. Tumour mass dormancy occurs when the rate of cancer cell proliferation is balanced with cell death, causing no net change in tumour volume [1] and encompasses angiogenic and immune-mediated tumour dormancy (see review [2]). This definition evolved from the original theory of ‘population dormancy’ proposed in 1972 [3]. In comparison, cancer cell dormancy refers to individual cell(s) in reversible cell cycle arrest, that are capable of reactivation and tumour expansion [4], and will be the focus of this review.

**Fig. 1:**
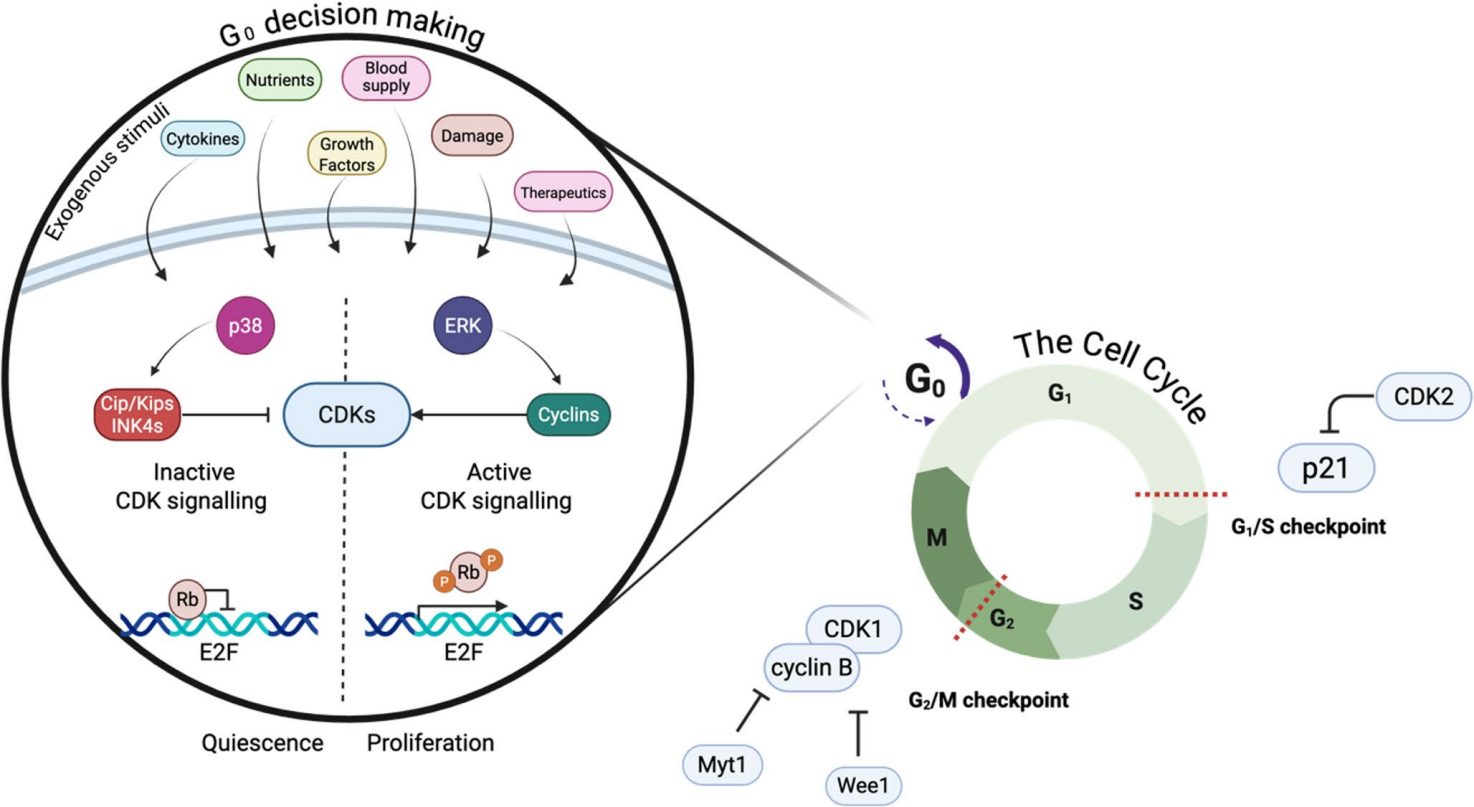
Cell cycle control in dormancy. Cellular dormancy is governed by a number of signalling pathways which decide whether the cell enters or exits G_0_. These decisions are influenced by external factors such as cytokine signalling from surrounding cells, nutrient depletion, changes in blood supply, growth factors, damage inducers and therapeutics. These can affect the balance of Cyclin-dependent kinase (CDK) signalling, which is a key regulator of cell cycle progression. Increased p38 levels can lead to activation and accumulation of CDK interacting proteins/kinase inhibitory proteins (Cip/Kips) and CDK4 inhibitors (INK4s) These inhibit CDKs and contribute to quiescence by maintaining retinoblastoma protein (Rb) mediated inhibition of E2F transcription factor coding genes. In contrast, heightened levels of extracellular signal-regulated kinases (ERK) boosts cyclin activity which acts to promote proliferation via hyperphosphorylation of RB. In order to progress from G_1_ to S phase, the checkpoint needs to be passed. If p21 levels are too high, the cell is arrested. Similarly, the G_2_/M checkpoint must be passed by accumulation of the CDK1/cyclin B complex for the cell to start division. Signals that can act to prevent this include myelin transcription factor 1 (Myt1) and Wee1. Created in BioRender. Green, A. (2025) https://BioRender.com/nuy9a51

Dormant cancer cells are non-cycling and thus share traits with healthy quiescent cells, senescent cells and to a lesser extent differentiated cells, and other somewhat synonymous terms including diapause-like, drug-resistant persister cells and cancer stem cells. The similarities and differences are well-described in previous reviews by Risson *et al*. [2] and Weston & Barr [5]. The key difference between dormancy and senescence is that cell cycle arrest in dormancy and quiescence is reversible, whereas the senescent state is considered permanent. Cellular dormancy can be triggered by environmental stresses and is widely considered to be a protective mechanism to encourage survival in the metastatic niche (Fig. 1). Senescence, an age-related stress response, can trigger cell cycle arrest to protect against further damage. Senescence in aging is initiated by telomere shortening, which triggers the DNA damage response to prevent any further replication from occurring and exacerbating damage, via its activation of ATM: HK2 and p53:p21 [6]. The senescent state can also be induced by a variety of stimuli (e.g. irradiation [7]), and the senescence-associated secretory phenotype (SASP) of a cell can be influenced by these factors. Senescence within cancer has been linked to immune evasion, as these cells can help to reprogram the immune landscape of the tumour microenvironment [8]. Similarly, there is overlap between cancer stem cells and dormant cells, but not all dormant cells display stem cell-like characteristics [4]. Moreover, while dormant cells survive chemotherapy, many treatments also induce a dormancy-phenotype but there are likely differences in dormancy regulation between these two scenarios. The shared traits and markers (e.g. Ki-67-, p21+, p27+) between dormant, senescent and similar cells can make them difficult to distinguish and can sometimes lead to the terminology for these cells being interchanged.

## Mechanisms of Cancer Cell Dormancy

Cancer cell dormancy is observed across cancer types and tissues, with the bone microenvironment in particular containing pro-dormancy niche(s), and the ability to trigger reactivation [4, 9]. The bone microenvironment is required for dormant cells to persist over long time periods and offers protection from drug treatments and immune surveillance.

Dormancy should also be thought of as a dynamic ‘on-off’ process, where cells can enter dormancy, reactivate and divide, then their progeny can re-enter dormancy. With dormant cells retaining the same capacity as proliferating cells to repopulate tumours in myeloma [10]. Cells can remain dormant for long periods of time (months-years), but specifics around dormancy dynamics are poorly understood.

## Microenvironment Cell Regulation of Dormancy

**Fig. 2:**
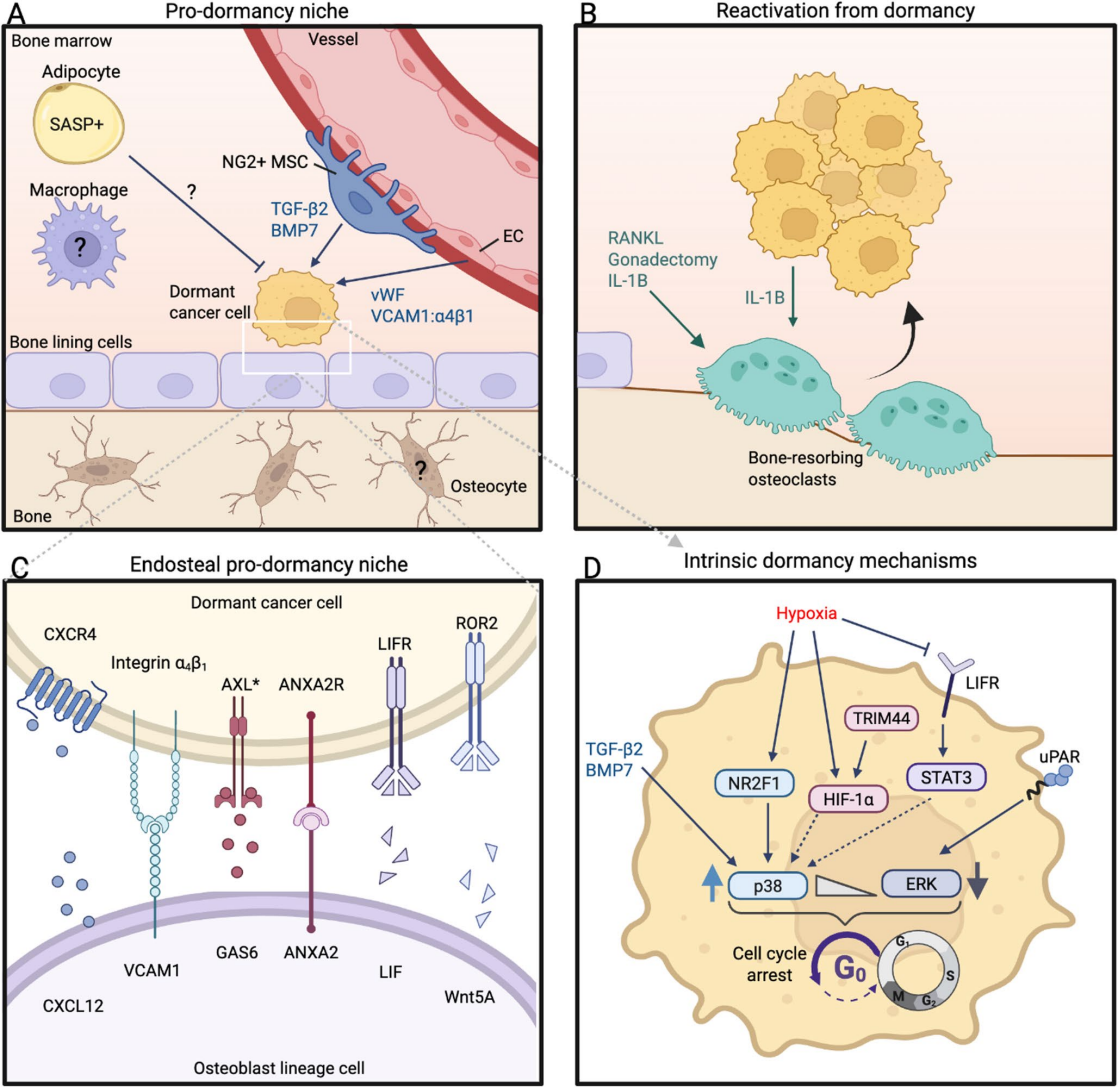
Extrinsic and intrinsic control of cancer cell dormancy in bone. **A **Dormant cancer cells reside in pro-dormancy niches, on the endosteal niche near bone lining cells and in breast cancer the perivascular niche. Proximal to endothelial cells (ECs) perivascular NG2 + Nestin + mesenchymal stromal cells (MSCs) promote dormancy via transforming growth factor (TGF)-β2 and bone morphogenetic protein (BMP)7. The role of immune cells, adipocytes and osteocytes are not yet well defined. **B **Reactivation can be triggered by induction of osteoclastic resorption by receptor-activated nuclear factor kappa-B ligand (RANKL), gonadectomy or interleukin (IL)−1B. **C **The endosteal pro-dormancy niche regulates dormancy via intercellular signalling pathways. **D **Cell-intrinsic dormancy pathways. C-X-C chemokine receptor type 4 (CXCR4), CXC ligand 12 (CXCL12), vascular cell adhesion molecule 1 (VCAM1), growth arrest-specific 6 (GAS6), annexin A2 (ANXA2) receptor (ANXA2R), leukaemia inhibitory factor (LIF) receptor (LIFR), receptor tyrosine kinase-like orphan receptor 2 (ROR2), tripartite motif-containing protein 44 (TRIM44), nuclear receptor subfamily 2 Group F member 1 (NR2F1), hypoxia-inducible factor (HIF), signal transducer and activator of transcription (STAT), extracellular signal-regulated kinase (ERK), urokinase plasminogen activator receptor (uPAR), von Willebrand factor (vWF). Created in BioRender. Green, A. (2025) https://BioRender.com/jndnuas

Cancer cell dormancy is controlled by extrinsic signals from the microenvironment (Fig. 2). Throughout the bone marrow there are many different cell types that form specialised niches, each capable of performing unique tasks that contribute to haematopoiesis, bone maintenance and other homeostatic functions. Cancer cells take advantage of these niches, exploiting them to promote cancer cell persistence, survival and drug resistance. Many of the well-established mechanisms are discussed in a thorough review by Risson *et al*. [2] and we have focused on recent advances in the dormancy and senescence niche paradigm.

In bone marrow cancers, like myeloma and leukaemia, the primary site is the bone marrow, but these cancers still spread through multiple skeletal sites. In bone metastatic cancers, disseminated tumour cells (DTCs) will leave the primary tumour and spread via the circulation to bone. While many different cancer types grow in or spread to bone [11], the dormancy mechanisms have many similarities across cancer types despite the tissue of origin. In metastatic breast and prostate cancers, DTCs can be detected in the bone prior to overt metastasis [12–15]. Cancer cells can lie dormant for long periods of time, often causing the disease to return after years of remission for patients who achieved a ‘complete response’ (i.e. no detectable disease), or minimal residual disease (MRD [16–18], i.e. very low, usually stable, levels of disease). Dissemination to the bone is an inefficient process, only a small proportion of cells survive and repopulate tumours. However, we do not yet know whether dormancy is an entirely microenvironment-dependent process where chance engagement with the pro-dormancy niche is random, or whether the surviving dormant cells already exhibited intrinsic factors that encourage the dormancy-survival process. Moreover, the dynamics of dormancy in the bone marrow have not yet been characterised in detail *in vivo* in terms of duration of quiescent periods, the frequency of switching between quiescent and cycling state, and whether dormancy dynamics vary across sites in the skeleton. Given different skeletal sites have different niche and blood cell compositions [19, 20], and different responses to stress [20], it seems plausible that there could be site heterogeneity in single cell cancer dormancy dynamics in bone. The bone also reprogrammes DTCs for further spread to other soft tissue and skeletal sites. In bone metastatic breast and prostate cancer, enhanced activity of the histone methyltransferase enhancer of zeste 2 (EZH2) reprogrammes disseminated cells into a more stem-like phenotype, and elevates their capacity to seed other sites in mice, which can be prevented with an EZH2 inhibitor [21]. A related finding in patients is that endocrine therapies can induce epigenetic modifications, but not genetic alterations, that promote dormancy induction [22]. This indicates that dormancy is heavily influenced by extrinsic stimuli, and not a consequence of acquisition of mutations that promote a dormant state.

The bone marrow recruits cancer cells via chemoattractants usually involved in healthy haematopoiesis. This includes the C-X-C chemokine ligand 12 (CXCL12)-CXC receptor 4 (CXCR4) chemokine axis which recruits and retains early B lymphocytes, plasma cells and haematopoietic stem cells (HSCs) in the bone marrow [23, 24]. DTCs typically engage in the endosteal and/or perivascular niches [10, 24–27] when they arrive in the bone marrow, where they can be maintained in a dormant state for extended periods of time. Generally, the mesenchymal/osteoblast lineage have most consistently been implicated in promoting/maintaining dormancy in the bone microenvironment across different cancer types [10, 23, 26–30], although whether there is one pro-dormancy niche, or multiple niches and the specific cell type(s) involved is an ongoing area of research (Fig. 2A). Dormant cells have been shown to reside near type I collagen (Col2.3 GFP+) [10], osteopontin [23, 26] and ALCAM-expressing cells [27, 28] on the endosteal surface, and osteoblasts protected cancer cells from oxidative damage [26] and hypoxia [28]. Bone lining cells are heterogeneous cell populations [19], and the pro-dormancy niche is likely a particular subset of these cells, as has been shown for the pre-B lymphocyte niche which is supported by AB bone lining cells (Lin-CD31-CD51+Sca-1-PDGFR⍺+PDGFRβ+) [19]. In breast cancer, dormant cells are also in close proximity to endomucin+ perivascular niches [23] and specifically regulated by NG2+Nestin+ mesenchymal stromal cells (MSCs) [31]. The perivascular niche is protective to breast DTCs in the bone marrow [32, 33] providing integrin-mediated resistance to chemotherapy, irrespective of whether they are dormant (p27+) or cycling (p27-), and targeting endothelial-derived von Willebrand factor and vascular cell adhesion molecule 1 (VCAM1) sensitised mice to chemotherapy to prevent bone metastases [33]. Recently, bone metastases with different primary origins were shown to display one of three distict immune ecosystems, that were enriched for either macrophages & osteoclasts, monocytes, or regulatory & exhausted T cells [34]. The dormancy niche was not studied, but dormancy is likely influenced by these unique enrichments of different immune cell types.

The bone niche produces several pro-dormancy factors (Fig. 2A, C) that enable niche engagement (e.g. CXCL12) and maintenance of dormancy [e.g. growth arrest-specific 6 (GAS6) [29, 35], bone morphogenetic protein 7 (BMP7) [31, 36], transforming growth factor (TGF)-β2 [31, 37], leukaemia inhibitory factor (LIF) [38]. A pathway that is implicated in dormancy maintenance across multiple cancer types in bone is GAS6 expressed by osteoblast lineage cells and also breast cancer cells [39], binding to TYRO3, AXL or MER (TAM) tyrosine kinase receptors, although the type of receptor expressed varies across cancer types. The AXL-Gas6 axis is important in myeloma and prostate cancer cell dormancy [29, 40, 41], and similarly the MER-GAS6 axis promotes dormancy in acute lymphoblastic leukaemia [42]. AXL inhibitor treatment in mice, reduces the proportion of dormant myeloma cells and increases tumour burden, suggesting disruption of the AXL-GAS6 axis inhibits dormancy and thereby triggers reactivation [29]. In breast cancer, TGF-β2 and BMP7 derived from NG2+/Nestin+ MSCs maintains dormancy. Indeed, deletion of periarteriolar NG2 + cells using inducible or conditional deletion of TGF-β2 in mice prior to intracardiac injection of E0771-GFP breast cancer cells, reduced the number of p27 + dormant cells and increased prevalence of bone metastasis [31]. While these studies show disruption of AXL in myeloma [29], or NG2 + MSC TGF-β2 expression can reactivate dormant cells, not all cells were awakened. It is now evident that a multitude of factors are implicated in the pro-dormancy niche, meaning dormancy maintenance is a multi-factor process and is not completely dependent on any one pro-dormancy signal. In breast cancer, Ren *et al*. identified genes upregulated by dormant cells isolated from the bone of mice with PyMT-B01 cells [39], and their dormancy gene signature correlated with better outcomes in patients. However, knockdown or overexpression of each of these genes (*Cfh*,* Gas6*,* Mme*,* Ogn*) individually in breast cancer cells had no measurable impact on tumour burden when implanted into mice, although dormant cell numbers were not reported in this study. AXL knockout in prostate cancer cells does not alter dormancy induction [41]. While this possibly indicates that each of these genes are a consequence but not cause of dormancy, they also support the notion that dormancy is dependent on the niche, and cell dormancy is a multi-factor process with built-in redundancy.

Dormant cells can be reactivated by signals that trigger cell cycle re-entry (Fig. 2B). In bone, a well-established mechanism of dormancy exit in myeloma, breast and prostate cancer is through stimulation of osteoclastic resorption. This can be induced with the osteoclastogenic factor receptor-activated nuclear factor kappa-B ligand (RANKL) [10], castration [43] or ovariectomy [44], which can be inhibited with anti-resorptives zoledronic acid or OPG-Fc [45]. A recent trial demonstrated adjuvant zoledronic acid reduced DTCs in breast cancer patients with DTC-positive bone marrow [46]. Currently, the molecular mechanism of reactivation following resorption is unclear, but could be due to release of growth factors from the bone matrix that promote cell cycle re-entry, or disruption of the pro-dormancy niche. Microenvironment-derived interleukin (IL)−1B also promotes development of breast cancer bone metastases in mice via Wnt signalling [47–50]. Interestingly, tumour and microenvironment-derived IL-1B has the opposite effect in the primary tumour, where infiltration of anti-tumour immune cells impairs tumour growth. Inhibition of IL-1B with anakinra could prevent development of bone metastases when combined with zoledronic acid and doxorubicin [47]. In breast cancer, micrometastases form in the osteogenic niche near active osteoblasts [25], and the cancer cells rely on transfer of calcium via connexin 43 gap junctions [51]. Therefore, a change in tumour-microenvironment signalling within the pro-dormancy niche can initiate the switch from quiescence to cell cycle re-entry.

Recent developments have identified that a senescent microenvironment promotes progression and proliferation in myeloma. Myeloma cells induced a senescence-like phenotype in bone marrow adipocytes, which in turn induced resistance to dexamethasone [52]. Myeloma is preceded by a premalignant condition monoclonal gammopathy of undetermined significance (MGUS), with a yearly 1% risk of progression from MGUS to myeloma. In patients with senescent MSCs the risk of progressing from MGUS to myeloma was higher. Senescent (β-galactosidase+) bone marrow MSCs promoted proliferation of myeloma cells, through upregulation of senescence factors including Gremlin-1 [53]. While an aged, senescent phenotype of microenvironmental cells promotes progression of myeloma, this may not be the case when the senescent phenotype is exhibited by plasma cells. Borges *et al*. recently showed that plasma cells from MGUS and myeloma patients exhibit a senescence-like phenotype based on several senescence gene lists compared to healthy plasma cells [54]. The senescence phenotype was higher in patients with stable MGUS compared to progressive disease [54], aligning with other studies showing the SASP phenotype aids in clearance and cellular turnover via immune activation [55]. An interesting finding was that the senescent plasma cells had a paracrine effect inducing senescence in surrounding microenvironmental cells [54]. It should be noted that, the senescence traits have considerable overlap with traits of dormant cancer cells (e.g. p21+, Ki-67-), and dormancy gene sets similarly show survival advantages in patients [29, 39].

## Extrinsic Dormancy Factors

Beyond cellular interactions, extrinsic factors of the tumour microenvironment can induce or regulate dormancy including extracellular matrix (ECM) composition, hypoxia, nutrient availability and therapeutics (Fig. 2D). These are common methods for inducing dormancy *in vitro* [56], but not all these mechanisms have been studied and validated in the bone microenvironment *in vivo*. For instance, the bone microenvironment contains a complex ECM. Yet while the ECM controls dormancy at other sites [57–60], and integrins are important for bone metastasis [61], it is not clear whether the bone ECM directly regulates dormancy.

Oxygen levels regulate cellular metabolism and control of quiescence and proliferation. Compared to normal tissues (2% − 9%), the concentration of oxygen in the bone marrow is low (hypoxic, < 1% − 6%) [62]. Hypoxia is critical for the maintenance of quiescence in healthy stem cells, and hypoxia also supports cancer stem cells and chemotherapy resistance [63]. In myeloma, hypoxia induces stem cell-like features [64, 65] and stabilisation of hypoxia inducible factor (HIF)1α by the deubiquitinase tripartite motif-containing protein 44 (TRIM44) maintains quiescence *in vivo* [28]. In breast cancer, hypoxia induces dormancy in the primary site and in DTCs, and cells remain dormant even once hypoxia is removed [66], however prolonged hypoxia downregulates LIF receptor (LIFR) triggering escape from dormancy and formation of bone micrometastases [38] (Fig. 1D).

Cancer therapeutics themselves can also induce dormancy in multiple cancers. In myeloma, standard of care treatments bortezomib or melphalan have been observed to induce a dormancy-phenotype in surviving bone-resident cells [67, 68]. These findings highlight the complex challenge of eliminating all cancer cells to ensure relapse prevention.

## Intrinsic Dormancy Control

Intrinsically, cellular replication or quiescence/arrest decisions are tightly governed by the presence of CDKs and their associated cyclins (Fig. 1) [69]. Specific thresholds of cyclins and CDKs are required to pass each cell cycle restriction point to allow cell cycle entry and proliferation [70]. Specifically, increased cyclin-D increases CDK4/6 which phosphorylate the transcriptional repressor Rb and enable expression of downstream proliferation factors [71]. Conversely, reduced CDK activity confers normal quiescence and cancer dormancy [5]. A large variety of intra- and extra-cellular signalling pathways [9, 72, 73], converge on CDK regulation to allow dynamic cell cycle control in response to cellular conditions. The cyclin D/CDK4/6–Rb protein pathway is critical to the proliferation of both normal and malignant breast epithelial cells [74]. Inhibiting CDK4/6 with FDA-approved agents (e.g.abemaciclib, palbociclib [75], and ribociclib) combined with hormonal therapy, has proven effective in treating HR+ metastatic breast cancer [9] by preventing Rb phosphorylation and inducing G_1_ cell cycle arrest [74], supporting the role of CDK4/6 in dormancy regulation. Regardless of stimuli, cancer dormancy pathways largely intersect on p38 and ERK regulation which respectively decrease CDKs and promote cyclin expression (Fig. 1) [76], with this balance controlling dormancy maintenance and release. The maintenance of this anti-proliferative signalling is essential to enable the long-term viability of dormant cancer cells.

### Genetic, Epigenetic and Transcriptomic Alterations

Genetic heterogeneity is conserved within proliferative and dormant drug-tolerant states [77], and instead dormancy decisions are determined by epigenetic and transcriptional alterations. Evidence from breast cancer shows stochastic entry and asynchronous exit from dormancy under uniform conditions [22], reinforcing the theory that dormancy dynamics are driven by epigenetic and transcriptional reprogramming.

Epigenetic regulation is largely controlled by histone methylation and acetylation which dictate accessibility of the DNA to control transcription [78]. Use of histone deacetylase inhibitors (HDACi) in breast cancer, can induce LIFR and consequently pro-dormancy mechanisms [38, 79, 80]. Similarly, in prostate cancer HDACi repress metastatic outgrowth in bone through restoration of interferon signalling [81]. HDACi are also approved for use in myeloma, but the effect on dormant cells has not been investigated. Moreover, the histone methyltransferase Smyd5 maintains breast cancer dormancy, and similarly the histone methylation inhibitor 5-azacytidine facilities maintenance in breast cancer and head and neck squamous cell carcinoma (HNSCC) [82, 83]. A combination of 5-azacytidine and all-*trans* retinoic acid (ATRA) increased expression of nuclear receptor subfamily 2 Group F member 1 (NR2F1) maintaining dormancy via SOX9 and retinoic acid receptor (RAR)β in prostate cancer and HNSCC [84, 85].

Dormant cancer cells in the bone microenvironment exhibit a unique transcriptome compared to their proliferating counterparts. In myeloma, single cell RNA sequencing revealed dormant cells (DiD^hi^, 5TGM1 mouse model) express a transcriptome signature that is more akin to myeloid lineage cells including monocytes and macrophages [29]. In prostate cancer bone metastases, dormant cells (PGH^+^, RM1 mouse model) were enriched for genes involved in type I interferon signalling, and loss of this signature led to overt bone metastases [81]. Inflammatory and immune signatures are also enriched in dormant breast cancer cells (DiD^+^ cells, PyMT-B01 mouse model) [39]. Thus, while not identical, there are several genes and pathways that are consistently altered in dormant cells across cancer types, including the AXL-GAS6 axis. The precise mechanism that switches on these gene signatures has not yet been fully elucidated, but is thought to be microenvironment induced, and various microRNAs have also been implicated in transcriptional regulation and dormancy induction [86, 87].

### Signalling Mechanisms and Metabolism

Pro-dormancy cues ultimately lead to induction and long-term maintenance of cell cycle arrest. Several pathways (e.g. TGF-β2:TGBRIII, BMP7:BMPRII [40], LIF: LIFR [38]) converge at central dormancy-proliferation mediators, such as p38, ERK and STAT3/AKT to enable long-term dormancy survival (Fig. 2D). Downregulation of urokinase-type plasminogen activator receptor (uPAR) has been shown to reduce ERK signalling and drive dormancy maintenance through inhibition of the Src kinase/focal adhesion kinase pathway [88, 89], and *in vivo* targeting of Src and MEK1/2 prevented reactivation and metastasis [90, 91]. However, to date there has been little progress in therapeutic approaches targeting these signalling axes for cancer dormancy within bone.

Dormant cancer cells are dependent on autophagy, a process of cellular recycling of dysfunctional or unnecessary organelles and proteins [92]. Given dormancy can be a response and route to survive therapy, autophagy may offer a mechanism for cells to recover from oxidative/metabolic stress. Targeting autophagy, and in particular mitophagy, with hydroxychloroquine or knockdown of autophagy-related gene 7 (ATG7) caused accumulation of dysfunctional mitochondria and oxidative stress sufficient to induce apoptosis [92], highlighting a novel therapeutic avenue. Similarly, autophagy induction through increasing tumour suppressor aplasia Ras homolog member I (AHRI) in ovarian cancer promoted dormancy [93]. While these studies have not focused specifically on bone, autophagy is likely a general intrinsic stress-response mechanism, and not microenvironment-specific.

Pancreatic endoplasmic reticulum kinase (PERK) has also been identified as a regulator of survival in tumorigenic and “spontaneous” dormant human epidermoid carcinoma HEp3 cells both *in vitro* and *in vivo* [94, 95]. While PERK is significantly involved in the unfolded protein response (UPR) [96], survival of dormant cells is likely facilitated through its functions in autophagy and oxidative stress regulation [97]. Quiescent cells undergo metabolic shift away from glucose dependency to favour oxidative phosphorylation elevating ROS production, often induced by hypoxia [98, 99]. The master antioxidant transcription factor NRF2 is a direct target of PERK. PERK deletion caused inactivation of NRF2, resulting in attenuated growth and increased oxidative stress [100]. As such the dual protective role of PERK has been proven significant in dormant cancer cell survival [94, 95]. Calvo *et al*. (2023) recently showed selective *in vivo* depletion of HER2 + breast cancer DTCs in bone marrow and lung with PERK inhibitor HP40 treatment [101]. This was mirrored using spontaneously dormant D-HEp3 HNSCC cells [101], highlighting PERK as a possible novel therapeutic target for dormant cell specific toxicity.

## Clinical Trials Targeting Cellular Dormancy in Bone Metastatic Cancers

**Fig. 3:**
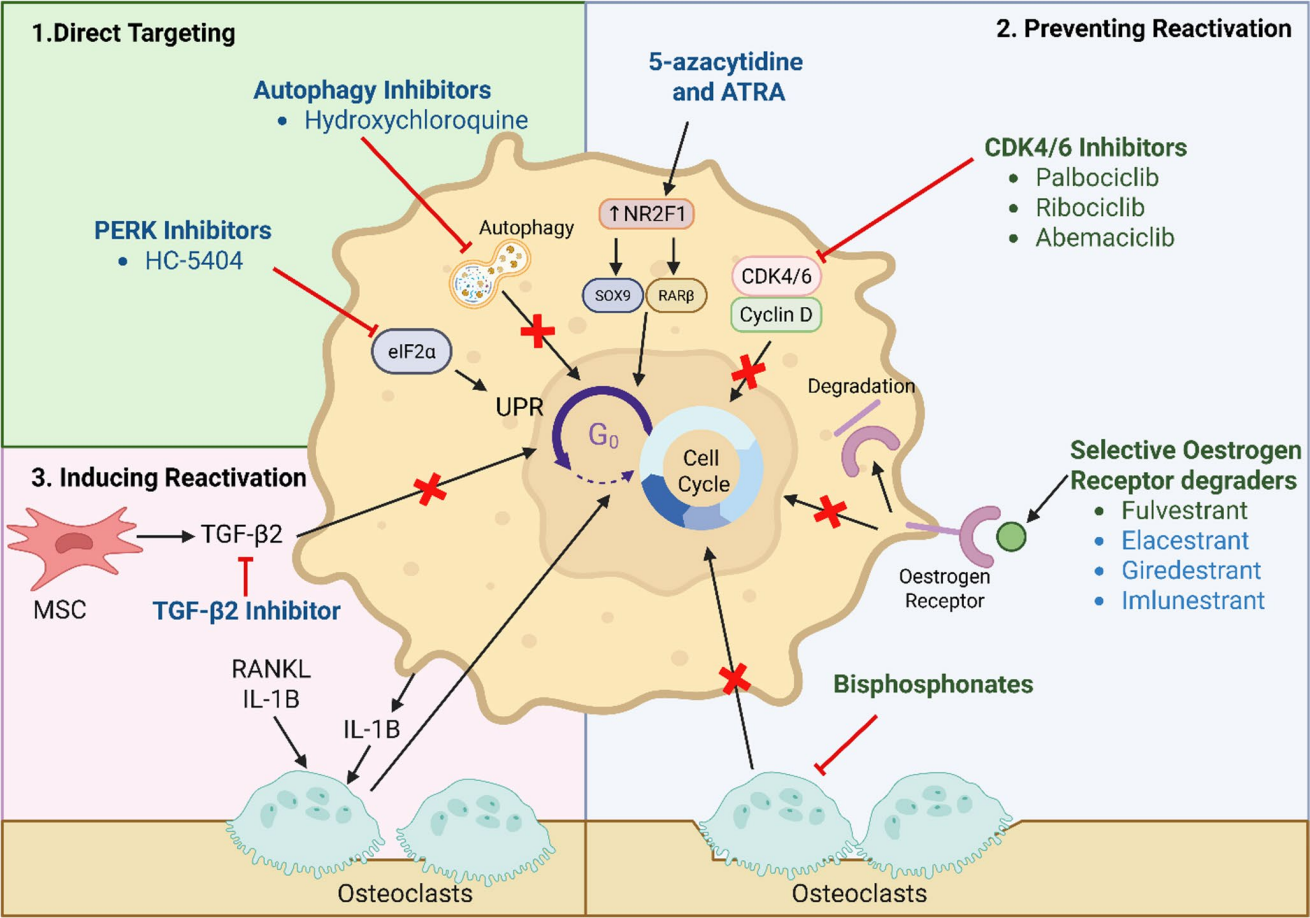
Strategies to target dormant cancer cells based on dormant cell biology. **1 **Directly targeting dormant cancer cells offers a strategy to completely eliminate dormant residual disease. Autophagy inhibitors and/or PERK inhibitors are promising therapies which disrupt survival mechanisms that dormant cells rely on during metabolic stress (autophagy and the unfolded protein response (UPR), respectively) [92, 101, 105–107]. **2 **Preventing reactivation of dormant cancer cells offers a strategy to maintain dormancy and prevent relapse. CDK4/6 inhibitors, selective oestrogen receptor degraders (SERDs), bisphosphonates and 5-azacytidine and all-*trans* retinoic acid (ATRA), are promising/approved therapies that interfere with key pathways involved in dormancy, such as inhibiting cell cycle re-entry [108–114], epigenetic reprogramming via NR2F1 via SOX9 and RARβ [84, 85], and inhibiting the supportive osteoclastic resorption that induces tumour proliferation in the bone microenvironment [46]. **3 **Inducing reactivation of dormant cancer cells offers a strategy to induce the dormant cell population into a proliferative cell population that is more susceptible to standard chemotherapeutic treatment. Strategies such as using RANKL and IL-1B to stimulate osteoclastic resorption [10, 48–50], and TGF-β2 inhibitors which block pro-dormancy signals from mesenchymal stromal cells (MSCs) [40], both are promising strategies to reactivate the dormant cells back into the cell cycle. Therapies in green are approved, therapies in blue are in preclinical or clinical trials. Created in BioRender. Green, A. (2025) https://BioRender.com/spmuz42

Advancements in our understanding of dormancy biology has led to development or redeployment of several therapeutics in clinical trials (Table 1). Current strategies to eliminate dormant cancer cells fall into three main categories: (1) directly targeting dormant cells, (2) preventing reactivation, or (3) inducing reactivation to sensitize them to therapies targeting proliferating cells (Fig. 3). The latter approach is considered risky, as even a small subset of treatment-refractory/resistant proliferating cells could potentially drive incurable recurrent disease. As yet, there are no NICE or FDA-approved strategies that directly target dormant cancer cells, although some therapies can impair reactivation.

**Table 1:** Therapies targeting cancer cell biology in clinical trials. Original table

Drug	Clinical trial	*n*	Patient characteristics	Study design	Primary endpoints	Ref.
Palbociclib	Phase II Completed	60	RB + mHSPC	Palbociclib plus ADT vs. ADT	PSA RR after 28 days	[124]
Palbociclib	Phase II Active-not recruiting	-	mCRPC	Palbociclib	Clinical benefit rate, CR, PR, SD	(*NCT02905318*)
Palbociclib	PALOMA-2 Phase III Completed	666	postmenopausal women, ER+, HER2- MBC, no prior treatment for MBC	palbociclib plus letrozole vs. placebo plus letrozole	PFS assessed by the investigators	[116, 117]
Palbociclib	PALLAS Phase III Completed	5,761	ER+, HER2- EBC	Palbociclib plus adjuvant ET vs. adjuvant ET	iDFS	[120]
Palbociclib	PENELOPE-B Phase III Completed	1,250	ER+, HER2- High risk primary BC without a response to Taxine containing neoadjuvant chemotherapy	Palbociclib plus ET vs. placebo plus ET	iDFS	[121]
Abemaciclib	monarchE Phase III Ongoing	5,637	ER+, HER2-, node + high risk EBC	Abemaciclib plus ET (physicians’ choice) vs. ET (physicians’ choice)	iDFS	[118, 140]
Abemaciclib	CYCLONE 2 Phase III Active not recruiting	393	mCRPC, measurable disease and radiographic progression	Abemaciclib plus abiraterone and predniso(lo)ne vs. Placebo plus abiraterone and predniso(lo)ne	rPFS assessed by the investigators	[123]
Ribociclib	Phase Ib/II Completed	-	mCRPC chemotherapy naive RB + patients	Ribociclib plus enzalutamide	Maximum tolerated dose PSA Reduction	*NCT02555189*
Ribociclib	Phase Ib/II Complete	43 30 phase II	mCRPC in chemotherapy naive with progression on ARSI	Ribociclib plus docetaxel	6-month rPFS	[125]
Ribociclib	NATALEE Phase III Completed	5,101	ER+, HER2- EBC	Ribocicilib plus NSAI vs. NSAI	iDFS	[119]
Elacestrant	EMERALD Phase II Completed	477	MBC ER + HER2- with 1–2 prior lines of therapy, including CDK 4/6i	Elacestrant vs. standard of care (SOC) endocrine monotherapy (fulvestrant/AI),	PFS assessed by blinded independent central review (BICR)	[141]
Giredestrant	acelERA Phase II Completed	303	MBC ER + HER2- with 1–2 prior lines of therapy, including at least 1 ET	Giredestrant vs. physician’s choice ET	PFS assessed by the investigator in the overall population	[142]
Giredestrant	coopERA Phase II Completed	221	Untreated EBC and baseline Ki67 ≥ 5%	Window-of-opportunity phase with 14 days of giredestrant vs. anastrozole followed by 16 weeks of continued ET plus palbociclib	Ki67 change from baseline to week 2	[131]
Giredestrant	lidERA Phase III Active-recruiting	~ 4100	ER + HER2- Medium- and high-risk EBC	Giredestrant vs. physician’s choice of ET	IDFS	*NCT04961996*, [132]
Imlunestrant	EMBER-4 Phase III Active-recruiting	~ 6000	ER + HER2- EBC with adjuvant ET for 2–5 years and increased risk of recurrence	Giredestrant vs. standard ET	IDFS	*NCT05514054*, [133]
Hydroxychloroquine	Phase I Completed	14	ER + HER2- MBC	Hydroxychloroquine plus palbociclib plus letrozole	Safety and Tolerability Recommended phase II dose	[135]
Hydroxychloroquine	Phase I Active- not yet recruiting	TBC	Resectable, localised PC	Hydroxychloroquine vs. placebo	Change in expression of autophagy markers	*NCT06408298*
Hydroxychloroquine	CLEVER Phase II Completed-follow-up results to come		High Risk TNBC diagnosed within 5 years with positive nodes post completed neoadjuvant therapy except ET with detectable bone DTCs	Hydroxychloroquine or everolimus (mTOR inhibitor) or Hydroxychloroquine plus everolimus	Feasibility as defined at > 75% completion of C6C without G3/G4 AE	[143]
Hydroxychloroquine	ABBY Phase II Active- Recruiting	TBC	Histologically confirmed BC that has completed all primary treatments and has no evidence of recurrent local or distant BC.	Hydroxychloroquine plus abemaciclib vs. abemaciclib	Incidence of treatment-emergent adverse events Change in number if DTCs	*NCT04523857*
Hydroxychloroquine	PALAVY Phase II Active- Recruiting	TBC	ER + EBC with detectable DTCs in the bone marrow	Hydroxychloroquine or Avelumab (PD-L1 inhibitor) with or without Palbociclib	Proportion of subjects in each treatment arm with clearance of DTC at the end of the 6 cycles	*NCT04841148*
Hydroxychloroquine	Phase II Completed	52	PC with rising PSA after primary therapy- no radiographic evidence of metastasis and no ADT within 3 months	Hydroxychloroquine	PSA Response	[136]
Hydroxychloroquine	Phase II Completed	19	Oligometastatic PC (< 5 synchronous metastatic lesions) following primary tumour treatment	Hydroxychloroquine for 2 weeks prior to metastatic site directed radiotherapy	*≥* 50% induction of PAR-4 expression above baseline	[138]
HC-5404 (PERKi)	Phase Ia Completed	23	Advanced solid tumours	Dose escalation of HC-5404	*MTD*,* safety and tolerability*	[139]
AZA and atRA	Phase II Completed	14	PC post-local therapy with rising PSA (PSADT < 10 mo)	AZA & atRA vs. observation	safety and tolerability	[134]
Zoledronic acid	Phase II completed	45	BC Stage I-III with > 4 MM/mL DTC at baseline	Zoledronic acid	DTC and CTC measurement	[46]

*ADT *Androgen Deprivation Therapy; *ARSI *Androgen Receptor Signalling Inhibitors; *BC *Breast Cancer; *CTC *Circulating Tumour Cell; *DLT *Dose Limiting Toxicity; *DRFS*; *DTC *Disseminated Tumour Cell; *EBC *Early Breast Cancer; *ER *Oestrogen Receptor; *ET *Endocrine Therapy; *iDFS *Invasive Disease-Free Survival; *MBC *Metastatic Breast Cancer; *mCRPC *Metastatic Castrate-Resistant Prostate Cancer; *mHSPC *Metastatic Hormone Sensitive Prostate Cancer; *MTD *Maximum Tolerated Dose; *NSAI *Non-Steroidal Aromatase Inhibitors; *ORR *Overall Response Rate; *PC *Prostate Cancer; *PFS *Progression-Free Survival; *PSA *Prostate Specific Antigen; *RB *Retinoblastoma; *rPFS *Radiological Progression-Free Survival; *RR *Response Rate; *TBC *To Be Confirmed; *TNBC *Triple Negative Breast Cancer

A challenge in the development of therapies that target dormant cells is the need to reach every individual cancer cell, along with heterogeneity. Genomic analysis of samples across different skeletal sites in individual myeloma patients [102], or in melphalan-surviving clones [103, 104], indicate that relapse can be driven by a single clone and thus one dormant cell is sufficient to initiate relapse. As such, it is expected that complete elimination of dormant cells in all patients may require combination therapy in most patients.

## Therapies to Prevent Reactivation

Therapies that prevent reactivation of dormant cells can prolong survival by inhibiting relapse and metastatic outgrowth in bone. A number of strategies appear to be effective preclinically and have promising results in trials assessing bone metastasis.

### CDK4/6 Inhibitors

In advanced/metastatic breast cancer, CDK4/6 inhibitors (CDK4/6i) are approved in the UK for use in HR+ HER2- breast cancer, either in previously untreated patients or following endocrine therapy, in combination with fulvestrant or aromatase inhibitors [108, 109, 111, 112, 115]. The PALOMA-2 trial showed that palbociclib plus letrozole extended median progression free survival (mPFS) by 36.2 months and reduced disease progression risk by 59% in patients with low-burden bone-only disease compared to letrozole alone [116, 117]. In comparison, adjuvant trials in early breast cancer have shown only modest (monarchE [118]), NATALIE trials [119]) or no (PALLAS [120], PENELOPE-B [121]) improvement in survival outcomes. However, these trials resulted in the recommended use of adjuvant abemaciclib with endocrine therapy in HR+ HER2- early breast cancer at high risk of recurrence [122]. Guidelines regarding use of adjuvant ribociclib with an aromatase inhibitor for HR+ HER2- early breast cancer are soon to be announced [115]. The effectiveness of CDK4/6i at preventing bone metastatic outgrowth is likely via preventing dormancy reactivation, although has not been assessed clinically.

CDK4/6i are also in clinical trials for prostate cancer (Table 1), however these have not specifically assessed outcomes in the bone metastatic setting. Thus far, CDK4/6i have been well-tolerated when combined with other therapies, but clinical efficacy has not been strongly evidenced. The phase III CYCLONE 2 trial found no improvement in radiographic PFS by adding abemaciclib to abiraterone in castrate-resistance prostate cancer [123]. Similarly, a phase II trial found no radiographic PFS benefit over androgen deprivation therapy alone in Rb-positive metastatic hormone-sensitive prostate cancer [124]. Other combinations are in phase I/II investigation [125] (*NCT02555189*,* NCT02905318*), and given the promising efficacy in breast cancer bone metastasis, it is of interest to determine whether CDK4/6i could be similarly effective in prostate cancer bone metastasis.

### Selective Oestrogen Receptor Degraders

Oestrogen receptor (ER)-targeted therapies are crucial for treating ER-positive breast cancer and are used as long-term adjuvant therapies to prevent recurrence from dormant DTCs [126]. Selective ER degraders/downregulators (SERDs) have been developed to overcome endocrine resistance, particularly in *ESR1-*mutant breast cancers [113, 114], which results in the constitutive activation of ER and reduced sensitivity to standard oestrogen therapies (ETs) [113, 127]. SERDs could prevent reactivation by overcoming endocrine resistance in dormant DTCs. Currently, fulvestrant is the only FDA approved SERD, used in ET-refractory advanced/metastatic breast cancer [128–130]. New orally bioavailable SERDs (Table 1) show promising efficacy and tolerability in early and advanced breast cancer [128]. Early data from the coopERA trial showed a reduction in Ki-67 from baseline to week 2 in early breast cancer [131], suggesting SERDs may reduce tumour proliferation. Ongoing phase III trials are evaluating their potential in ER+ HER2- early breast cancer [132, 133].

### 5-azacytidine and ATRA

ATRA and 5-azacytidine has shown promise preclinically in dormant HNSCC and breast cancer [83, 84]. This motivated an ongoing clinical trial with 5-azacytidine and ATRA to induce dormancy and prevent relapse in biochemically recurrent prostate cancer patients. The combination appears safe and well tolerated, and a small subset of patients demonstrated clinical activity [134].

## Direct Targeting of Dormant Cancer Cells

Therapies that directly kill dormant cells could ultimately be curative if every dormant cancer cell is targeted. Some agents that target dormancy biology have shown promising results preclinically and are now in trials assessing bone metastasis.

### Hydroxychloroquine

Cancer cell dormancy is reliant on autophagy [92, 105–107], and the autophagy inhibitor hydroxychloroquine (HCQ) is in clinical trials to prevent recurrence in breast cancer specifically by targeting dormant DTCs (Table 1). HCQ is safe and tolerated with palbociclib in patients with metastatic breast cancer [135]. Ongoing PALAVY (HCQ or avelumab (PD-L1 inhibitor) ± palbociclib, *NCT04841148*) and ABBY (abemaciclib ± HCQ, *NCT04523857*) trials will determine efficacy in eliminating DTCs and prevention of disease recurrence. There are several prostate cancer trials [136–138] (*NCT06408298*), although these are not specifically assessing dormancy or bone metastasis.

### PERK Inhibitors

Preclinical studies have demonstrated the efficacy of PERK inhibition in HER-2 breast cancer [101]. The PERK inhibitor HC-5404 showed safety, tolerability and promising efficacy in a phase I trial in patients with advanced solid tumours [139]. However, further trials are needed to determine whether PERK inhibitors can effectively target dormant cells in bone.

## Conclusions

Recent technological advances, particularly in single cell omics, have rapidly advanced the dormancy field, uncovering key traits, mechanisms and potential therapeutic targets. Perivascular MSCs (NG2+ Nestin+) have emerged as pro-dormancy niche cells, yet the specific osteoblast-lineage subtypes and signalling pathways within the endosteal niche remain undefined, as does the likely complex role of immune cells. While dormancy-maintaining treatments are not curative, they can delay progression, and clinical trials are underway that exploit intrinsic vulnerabilities of dormant cells. To identify patients likely to benefit from dormancy therapies, efforts should focus on mapping conserved niche-cancer interactions across cancer types, and whether cellular heterogeneity enables persistence of resistant clones. With ongoing clinical trials, and new targets emerging from preclinical studies, the coming years are likely to reveal whether dormancy therapeutics can meaningfully benefit and extend the lives of patients with cancer in their bones.

## Key References

- Zhang et al. (2021) *The bone microenvironment invigorates metastatic seeds for further dissemination*. Cell.
  - This paper used evolving barcodes to show that the bone microenvironment reprogrammes disseminated breast and prostate cancer cells making them more likely to further metastasise to bone and other organs.
- Nobre et al. (2021) *Bone marrow NG2+/Nestin + mesenchymal stem cells drive DTC dormancy via TGF-β2*. Nature Cancer.
  - The paper identified that perivascular MSCs promote dormancy of disseminated breast cancer cells via TGF-β2 and BMP7. Disruption of this pro-dormancy niche led to a reduction in dormant cells.
- Vera-Ramirez et al. (2019) *Autophagy promotes the survival of dormant breast cancer cells and metastatic tumour recurrence*. Nature Communications.
  - The authors identify dormant breast cancer cells are sensitive to autophagy inhibitor hydroxychloroquine. This study has led to ongoing clinical trials using hydroxychloroquine and CDK4/6i to prevent relapse in breast cancer, with promising early findings.
- Khoo et al. (2019) *A niche-dependent myeloid transcriptome signature defines dormant myeloma cells.* Blood.
  - This paper revealed the single-cell transcriptome of dormant vs. proliferating myeloma cells mimics that of different blood cells - monocytes and macrophages. The dormant phenotype is dependent on the microenvironment and dormancy could be disrupted with inhibitors of AXL.
- Liu et al., (2025) Single-cell profiling of bone metastasis ecosystems from multiple cancer types reveals convergent and divergent mechanisms of bone colonization. Cell Genomics.
  - While not focused on dormancy, this paper used single cell RNA sequencing of 42 patient samples to reveal three distinct types of immune enrichment ecosystems in bone metastasis, and that these were not determined by primary cancer site.

## Data Availability

No datasets were generated or analysed during the current study.
